# Does Playing Mahjong Benefit Older Individuals? A Scoping Review

**DOI:** 10.14283/jpad.2024.102

**Published:** 2024-06-07

**Authors:** Z. C. K. Tse, Y. Cao, B. K. H. Chau, M. K. Yeung, C. Leung, David H. K. Shum

**Affiliations:** 1 Department of Rehabilitation Sciences, The Hong Kong Polytechnic University, Hong Kong, China; 2 Department of Social Work and Social Administration, The University of Hong Kong, Hong Kong, China; 3 University Research Facility in Behavioral and Systems Neuroscience, The Hong Kong Polytechnic University, Hong Kong, China; 4 Mental Health Research Centre, The Hong Kong Polytechnic University, Hong Kong, China; 5 Department of Psychology, The Education University of Hong Kong, Hong Kong, China; 6 School of Behavioral and Brain Sciences, University of Texas at Dallas, Richardson, TX USA; 7 Research Institute for Smart Ageing, The Hong Kong Polytechnic University, Hong Kong, China

**Keywords:** Leisure activity, mahjong, rehabilitation, healthy aging

## Abstract

Playing mahjong is a popular intellectual and social leisure activity in Asian countries. It is culturally believed that this activity is beneficial to cognitive and psychological functioning in older adults. However, empirical evidence of the benefits of playing mahjong is scant and scattered across the Western and Asian literature. This scoping review comprehensively examined previous studies of the relationships between playing mahjong and cognitive, psychological, and functional abilities in older adults, highlighted gaps in the literature, and identified directions for future research. A systematic search of the literature was conducted across thirteen Western and Asian databases. Fifty-three studies, including forty-seven observational and six intervention studies, were identified. Overall, the results of the observational studies suggested that more mahjong-playing experience was associated with better cognitive, psychological, and functional abilities. As an intervention, playing mahjong was found to enhance general cognitive abilities and short-term memory and relieve depressive symptoms. However, because most of the reviewed studies adopted a correlational methodology, the neural mechanism underlying the benefits of playing mahjong awaits further elucidation. The findings of this review suggest that more randomized controlled trials should be conducted to explore the effects of playing mahjong on higher-level cognitive functioning in older populations.

**Electronic Supplementary Material:**

Supplementary material is available in the online version of this article at 10.14283/jpad.2024.102.

## Does Playing Mahjong Benefit Older Individuals? A Scoping Review

**H**ow to maintain a good quality of life over the course of aging is receiving increasing attention. A growing body of research has focused on ways of promoting active aging, which emphasizes the optimization of health, participation, and security among older individuals. A potential method of promoting active aging is to foster participation in leisure activities, defined as activities in which people engage for enjoyment or to enhance their well-being rather than for work or as activities of daily living (1). Research has revealed that among older adults, participating in leisure activities could help to prevent physical and cognitive decline (2) and promote life satisfaction (3) and subjective well-being (4).

Mahjong, a traditional tile-based four-player game, is regarded as the national game in many Asian countries (5). A recent longitudinal study showed that from 2002 to 2018, almost a quarter (23.4% to 25.7%) of the Chinese population surveyed played mahjong or cards in their leisure time (6). Although mahjong playing may be associated with gambling issues in the past and put a heavy emphasis on good fortune to win the game, its popularity was advocated regardless of socioeconomic status, gender, and geographic areas (7). The game usually involves 136 to 152 tiles and requires players to take turns in drawing and discarding tiles until one of them claims victory by presenting a certain set of combinations (8). The literature on mahjong has classified the game as an intellectual and social leisure activity due to the complexity of its rules and because it involves multiple players (9). It is a cognitively and socially demanding activity that requires players to develop skills such as identifying potential matches, mentally retaining relevant information, deciding which tiles to discard, and predicting other players’ moves. Mahjong is regarded as a slow game in which players may plan the winning hands and revise the strategies simultaneously based on other players’ feedback (7). The uncertainties and luck components of the game may create pleasure feelings and attraction to the players, which promote and foster the popularity of the game. As a popular and culturally important activity in Asian countries, mahjong has the potential to concurrently enhance cognitive and psychological outcomes in older adults in Asian populations.

Emerging evidence has shown that playing mahjong could slow cognitive deterioration (10) and alleviate depressive symptoms in older adults with dementia (11). Mahjong players aged at 60 years or above have been found to show stable or even improved cognitive function (12), exhibit better eye–hand coordination than non-players (9), and experience a sense of being socially connected (13). Recognizing these potential benefits, a group of researchers in Hong Kong published a handbook of practical guidelines to promote and implement the game as an intervention in the community (14). Most of the literature on activities for older adults has focused on the benefits of culture-general activities such as reading, exercising, and playing memory games (15, 16). Some culture-specific activities, such as Tai-Chi and Qigong, have recently also become major topics of research (17). Considering the popularity of mahjong in Asian countries, research on playing mahjong, which is also a culture-specific activity, could make important contributions to this literature.

Although a growing body of evidence has indicated that older adults might benefit from interventions promoting mahjong, the literature has generally remained inconclusive in this regard. While the studies described above emphasized the benefits of playing mahjong, certain other studies have reported no or limited benefits associated with the activity in older adults (18, 19). Intervention studies have only partially supported the effectiveness of playing mahjong in achieving specific outcomes in older adults. For example, it has been found to support general cognitive performance and digit forward memory but not verbal or digit backward memory (8, 20). Therefore, it would be premature to conclude that playing mahjong improves general cognitive and psychological functioning in older adults. It is vital to review the results of previous studies to clarify which cognitive and psychological outcomes, such as memory, executive function, or psychological distress, are improved or alleviated by playing mahjong.

Evidence regarding the benefits of playing mahjong has been published in both the Western and the Asian literature. However, as many relevant Asian studies were written in Chinese, they have not been accessible to the wider research community; therefore, their findings have not been properly evaluated. This lack of access has made it challenging to synthesize the available evidence and identify directions for future research. Considering the popularity of mahjong in Asian countries, it is necessary to appraise the Asian literature together with the Western literature on this topic to enable a comprehensive understanding and interpretation of the findings. Only by summarizing, synthesizing, and analyzing all of the relevant literature is it possible to identify the key characteristics of playing mahjong that lead to its potential benefits.

In the current review, the evidence on this topic was mapped from a broad perspective using a scoping review approach. Specifically, we evaluated the existing literature, identified gaps, and determined directions for future research. The review addressed the following three research questions. (i) What are the main characteristics of studies of playing mahjong for older adults? (ii) What is the relationship between playing mahjong and cognitive, psychological, and functional abilities in older adults? (iii) What are the limitations of previous studies and potential future research directions?

## Methods

This scoping review was conducted and reported following the PRISMA extension for scoping reviews checklist (21), Arksey and O’Malley’s (2005) methodological framework, and the Cochrane Collaboration’s recommendations (22). The review was registered on the Open Science Framework (10.17605/OSF.IO/EDJ6G).

### Selection Criteria

Both English and Chinese empirical papers on playing mahjong were included to enable a comprehensive analysis and summary. Studies in which the participants’ mean age was 60 or above were included regardless of the participants’ health or clinical conditions. Studies that administered cognitive, psychological, and/or functional outcome measures were also included. There were no restrictions on the study design adopted in the selection criteria.

Studies that only reported the prevalence with which mahjong was played or focused on the problems associated with mahjong gambling were excluded. Studies that examined only physical outcomes, such as hypertension or mortality, rather than cognitive, psychological, or functional outcomes, were also excluded.

### Search Strategies

Systematic searches were conducted in the following Western and Asian databases from their inception to July 4, 2023: the Cochrane Library, PsycINFO, PubMed, CINAHL, EMBASE, the Web of Science, Scopus, CNKI, Duxiu Academic Search, Chinese Social Sciences Citation Index (CSSCI), NCL Taiwan Periodical Literature, Taiwan Citation Index—Humanities and Social Sciences (TCiHSS), and HKIChiP. Search terms were synonyms for “mahjong” and “older adults” and their translations in traditional and simplified Chinese (see Appendix A for an example). A university librarian was consulted for the database selection and keyword development, and the included Asian databases were representative and credible. The searches were not limited by language or date of publication.

### Screening and Data Charting

The searches were conducted by the first author and their results were imported to Covidence (https://app.covidence.org/) for title and abstract screening, full-text screening, and data extraction. The authors and research assistants worked in pairs that independently evaluated the titles and abstracts of the publications and completed the full-text screening and data charting on the co-developed data charting form. A consensus on article inclusion and data extraction was reached by discussion with a third reviewer when needed.

Data on sample characteristics (e.g., age, gender, years of education), the participants’ mahjong playing (e.g., frequency, duration, and experience), cognitive, psychological, and functional outcomes, and the details and efficacy of the mahjong interventions (e.g., content of the training programs and pre–post differences in both quantitative and qualitative outcomes) were extracted. The types of the studies and their findings were summarized to understand the relationships between playing mahjong and cognitive, psychological, and functional abilities in older adults.

## Results

**Figure 1:**
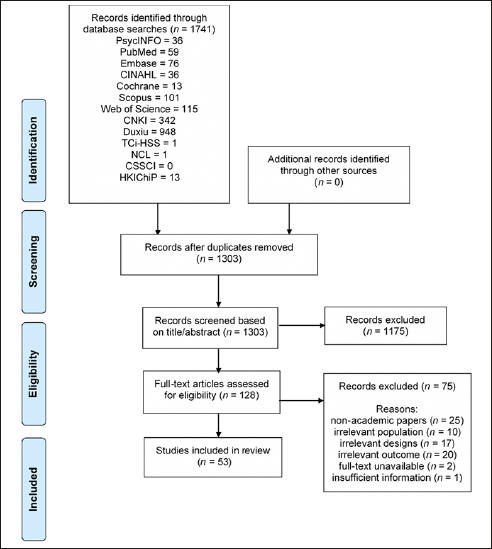
PRISMA Flow-Chart of the Study Selection Process

The literature search yielded 1,741 results in total. After eliminating duplicates, 1,303 unique records remained, and their titles and abstracts were screened. Of these, 128 records were retained for full-text screening. Fifty-three records were included in the final charting. The PRISMA flowchart in Figure 1 depicts the number of records included and the reasons for exclusion at each screening stage.

### Study Characteristics

Table S1 provides an overview of the characteristics of the reviewed studies. Most of the research was conducted in mainland China (n = 43, 81.1%). The remaining studies were conducted in Hong Kong (n = 7, 13.2%), Taiwan (n = 2, 3.8%), and New York (n = 1, 1.9%). Over half of the studies were in English (n = 30, 56.6%) and the remainder were in Chinese (n = 23, 43.4%).

The 53 studies were classified into two categories: observational studies (n = 47, 88.7%) and intervention studies (n = 6, 11.3%). Most of the observational studies utilized either a cross-sectional (n = 24, 51.1%) or a longitudinal (n = 16, 34%) design. The remainder adopted either a case-controlled design using clinical samples (n = 4, 8.5%) or qualitative interviews (n = 3, 6.4%). Among the six intervention studies, four were randomized controlled trials (RCTs; 66.7%) and two were non-RCTs (33.3%).

Most of the reviewed literature (n = 29, 54.7%) described playing mahjong as a general leisure activity and did not focus specifically on its cognitive or social characteristics in the research questions. The remaining studies considered mahjong an intellectual leisure activity (n = 10, 18.9%), a social leisure activity (n = 7, 13.2%), or “an intellectual and social leisure activity” (n = 7, 13.2%).

Another major characteristic of the studies was their widespread reference to mahjong in conjunction with card games and chess. Most of the studies defined mahjong as a general leisure activity, and these studies typically considered it together with card games and chess due to their similar nature. Specifically, the studies either investigated mahjong as an independent activity (n = 17, 32.1%) or considered it alongside card games (“mahjong or cards”; n = 27, 50.9%), chess (“mahjong or chess”; n = 1, 1.9%), or both (“mahjong, cards, or chess”; n = 8, 15.1%). Due to the limited number of studies on mahjong, excluding studies that combined it with card games and chess could have resulted in the loss of a significant proportion of the evidence. Therefore, instead of excluding those studies, we decided to include them and scope the mahjong literature and interpret the data cautiously. All of the intervention studies and qualitative studies investigated playing mahjong as an independent activity; only the observational studies grouped it along with the other two activities. The findings of this review remained qualitatively unchanged when the observational studies were excluded.

Among the cross-sectional, longitudinal and case-controlled studies (n = 44), 29 studies have reported the prevalence of mahjong playing among the population (M = 22.8%, SD = 9%, range = 2.6% to 42.9%). Apart from the dichotomous classification of mahjong play (yes/no), only 29 studies reported the ordinal frequency of mahjong playing in the population (e.g., everyday/weekly/ monthly/ occasionally/ never).

Only a few of the studies examined the effects of playing mahjong across different demographic groups. Generally, playing mahjong was a protective factor for cognitive impairment regardless of gender, geographic area (i.e., urban or rural areas), and years of education (6). One study found that males played mahjong with a higher frequency compared to females (19). However, other studies reported an equivalent effect of playing mahjong between males and females, in which playing mahjong was found to benefit cognitive ability (23), IADL (24) and promote successful ageing (25). Only one study reported significant age differences between playing mahjong and cognitive function, in which the effects were negligible for the participants aged 60–69 years old (26). However, some studies found that playing mahjong was a protective factor for cognitive impairment across all ages in older adults (23, 25). Most the studies focused on cognitive outcomes only (n = 31, 58.5%). Only a small proportion of the studies examined psychological (n = 10, 18.9%) or functional (n = 5, 9.4%) outcomes only. The remaining studies investigated multiple outcomes (n = 7, 13.2%).

The following subsections delineate the studies’ results according to their objectives, types of outcomes, and findings.

### Subjective Meaning Attached to Playing Mahjong

Three qualitative interview studies explored the benefits of playing mahjong by investigating its meaning for older adults in the community (5, 27, 28). Table S2 summarizes these studies’ findings regarding the meaning attached to playing mahjong for the participants and their experience of the activity.

Two of these three studies did not focus on a specific type of outcome (5, 28), whereas one study covered loneliness (i.e., a psychological outcome) (27). All of the studies agreed that playing mahjong provided benefits such as enhanced cognitive health and social and emotional support (5, 27, 28). The activity offered older adults a sense of competency and satisfaction and a feeling of youthfulness, and it also served as a form of mental exercise (5, 28). Additionally, it helped older adults to fill their leisure time and prevented social isolation (5).

### Short-term Benefits of Playing Mahjong

Thirteen cross-sectional studies examined the association between playing mahjong and cognitive, psychological, and functional outcomes. Table 1 summarizes the findings.

**Table 1:** Data Charting on Associations between Playing Mahjong and Cognitive, Psychological and Functional Outcomes (n = 13)

**Study**	**Country**	**Activity**	**Aim**	**Age**	**N**	**Main outcomes**	**Key findings**
Chou et al. (2004)	Hong Kong	Playing mahjong or cards	Relationships between leisure activity, socioeconomic and health characteristics	Aged 60 or above	2144	• Leisure activity participation • SPMSQ • BADLs and IADL • Self-rated health status, number of diseases, sight, pain	• Playing mahjong/cards was uncommon among Hong Kong older adults (did not play mahjong or cards = 74.9%). • Socioeconomic variables such as age, employment, being on welfare and IADL negatively associated with participation in mahjong/card playing. • Self-rated health and the number of diseases were positively associated with mahjong/card participation.
Zhou and Hu (2020)*	China	Playing mahjong	Relationship between intelligence games and cognitive function	Aged 60 or above	117	• Chess and card intelligence game participation • MoCA	Older adults who played mahjong (n = 3) showed better MoCA scores than those who did not (n = 114).
Fang and Shen (2017)*	China	Playing chess, cards, or mahjong	Relationship between playing chess, cards, or mahjong and cognitive function	Aged 60 or above	528	• Intellectual activity questionnaire • MoCA	Older adults who played mahjong/chess/card showed a higher score in MoCA compared to those who did not.
Liu (2020)*	China	Playing mahjong	Relationship between playing mahjong and cognitive function	Aged 60 or above	500	• Copeland’s Geriatric Mental State Examination • Simplified CSI-D	Playing mahjong was positively correlated with cognitive functioning.
Tsang et al. (2016)*	Hong Kong	Playing mahjong	Relationship between playing mahjong and eye-hand coordination	Aged 60 or above	41	• MMSE • EMG reaction time • EMG movement time • End-point accuracy of finger-pointing tasks	Compared with non-mahjong players, mahjong players showed better end-point accuracy in a finger-pointing test towards a stationary visual target on non-dominant hands; shorter EMG movement time on dominant hands and better EMG reaction time and end-point accuracy on non-dominant hands towards a moving target.
Ho and Chan (2005)	Hong Kong	Playing mahjong	Relationship between playing mahjong and cognitive function	Aged 55 to 88	204	• Leisure activities questionnaire • CDRS • IADL	Playing mahjong was negatively correlated with grip strength significantly, but it could not predict nor correlated with the CDRS score (i.e., the global cognitive functioning).
Ross and Zhang (2008)	China	Playing mahjong or cards	Effects of cognitively stimulating activities on the relationship between education and psychological distress	Aged 77 to 122	7944	• Frequency of Activities • Psychological distress • Years of formal education, Exercise, Self-reported health	Playing mahjong is associated with a low level of distress.
Yang et al. (2022)	China	Playing mahjong or cards	Relationship between social activities and depression	Aged 60 to 69	704	• GDS • Social engagement	Playing mahjong could not predict depression score.
Ren et al. (2021)	China	Playing mahjong or cards	Relationship between leisure activity and depression	Aged 65 or above	11727	• CES-D 10 • Leisure activities (yes/no)	Playing mahjong showed a significant negative association with depression, and playing mahjong daily is a protective factor against depression.
Zhao et al. (2023)	China	Playing mahjong or cards	Relationship between leisure activity and successful ageing	Aged 60 or above	7689	• Ascertainment of successful ageingt • Assessment of leisure activities (yes/no)	• Compared to older adults who never played mahjong, sometimes and usually playing cards or mahjong had greater odds for successful ageing • No age difference was found.
Wang et al. (2022)	China	Playing mahjong or cards	Relationship between mahjong or card playing and cognitive function	Aged 60 or above	7308	• Playing cards or mahjong (frequency) • MMSE	• There were statistically significant differences in MMSE total score and all subscales among the three types of frequencies in playing cards or mahjong, in which playing cards or mahjong regularly and occasionally had a significantly higher MMSE score compared to the non-participation. • Post hoc analyses showed age differences for the participants aged 70–79 and 80–80 years old but not for the participants aged 60–69 years old. • Playing cards or mahjong was the main component for explaining the MMSE score.
Mai et al. (2023)	China	Playing mahjong or cards	Relationship between social engagement, loneliness and cognitive functions	Aged 65 or above	12852	• Social engagement (frequency) • Loneliness (1-item) • Chinese version of MMSE	• Playing cards or mahjong was significantly positively related to cognitive function.
Ding et al. (2022)	China	Playing mahjong	Relationship between playing mahjong and MCI	Aged 60 or above	676	• Chinese version of MoCA • Playing mahjong (frequency and experience)	• Compared with the older adults with little mahjong experience (≤ 1 year), older adults with midlife mahjong experience more than one year were associated with reduced odds of having MCI. • The interaction terms “mahjong frequency and exercise” and “mahjong experience and exercise” were also associated with reduced odds of having MCI.

Note. SPMSQ = Short Portable Mental Status Questionnaire; BADL = Basic Activities of Daily Living; IADL = Instrumental Activities of Daily Living; MoCA = Montreal Cognitive Assessment Scale; MMSE = Mini-Mental State Examination; CDRS = Chinese version of the Mattis Dementia Rating Scale; EMG = Electromyography; CSI-D = Community Screening Instrument for Dementia; GDS = Geriatric Depression Scale; CES-D 10 = 10-item Centre for Epidemiological Studies Depression Scale; MCI = Mild cognitive impairment; ANO VA = Analysis of Variance; MANO VA = Multivariate analysis of variance. * Studies that have specified recruiting healthy older adults in the methodology. † Measures for ascertainment of successful ageing included self-rated health, CES-D-10, MMSE, activities of daily life, and physical activity.

Most of these studies focused on cognitive outcomes (n = 7, 53.8%; 18, 26, 29–33). Five of the studies investigated the effects of playing mahjong on psychological and functional outcomes such as depression, psychological distress, activities of daily living (ADL), and eye–hand coordination (38.5%; 9, 19, 34–36). One study (7.7%) created a composite variable named successful aging that incorporated cognitive, psychological, and functional measures (25).

Playing mahjong was positively related to general cognitive ability as measured by the Montreal Cognitive Assessment (MoCA; 29, 31), the Mini-Mental State Examination (MMSE; 32), and the simplified Community Dementia Screening scale (30). Compared with older adults who never played mahjong, those who were frequent and experienced players of mahjong exhibited better cognitive ability and reduced likelihood of having mild cognitive impairment (MCI; 26, 33). Three of the studies found that playing mahjong was negatively correlated with depression, psychological distress, and difficulties with ADL (34–36). One study reported that mahjong players had better eye–hand coordination than non-players (9) and another reported that mahjong players had a greater likelihood of successful aging, as measured by cognitive, psychological, and functional outcomes (25). One study reported no significant association between playing mahjong and dementia using the Chinese version of the Mattis Dementia Rating Scale (18), and another found no significant association between playing mahjong and depression (19).

### Long-term Benefits of Playing Mahjong

Sixteen longitudinal studies investigated the long-term benefits of playing mahjong. Table 2 summarizes the findings of these studies.

**Table 2:** Data Charting on Longitudinal Studies (n = 16)

**Study**	**Waves**	**Age**	**N**	**Activity**	**Construct(s)**	**Main outcomes**	**Key findings**
Xue (2020)	2002, 2005, 2008, 2011	aged 60 or above	2514	Playing mahjong or cards	Cognitive function	• MMSE • Playing cards or mahjong (yes/no) • Playing cards or mahjong (frequency)	• Compared with the older adults who did not play mahjong or cards, older adults who played mahjong or cards would report a higher MMSE score and slower decline over time. • Compared with the older adults who did not play mahjong or cards, older adults who played mahjong nearly every day, once a week, once a month, and seldomly showed a higher MMSE score. Playing at least once a month showed the largest effect, followed by playing mahjong every day, once a week, and seldom playing.
Ye et al. (2021)	2002, 2005, 2008, 2011, 2014	aged 65 or above	1040	Playing mahjong or cards	Cognitive function	• Chinese version of MMSE • Playing cards or mahjong (yes/no)	• Different patterns of cognitive decline showed different ratios of mahjong participation. There were significantly more older adults who played mahjong in the «medium and increasing group» than in the «high and declining group». • No significant difference was observed between the «high and declining» and «low and declining» groups.
Yi and Kang (2008)	1998, 2000, 2002	aged 80 or above	2251	Playing mahjong or cards	Cognitive function	• MMSE • Playing cards or mahjong (yes/no)	Older adults who played mahjong or cards showed significantly higher MMSE scores than those who did not. Two-level repeated measures analyses showed that playing mahjong / cards may significantly contribute to a higher level of cognitive functioning.
Yu et al. (2021)	2008, 2011/12, 2014, 2018	aged 61 or above	2439	Playing mahjong or cards	Cognitive function	• MMSE • Playing cards or mahjong (yes/no)	Compared with the «low initial level - cognitive decline group», there were more older adults in the «stable cognitive group» and the «high initial level - cognitive decline group» played mahjong/cards. The «high initial level - cognitive decline group» also showed a higher odd ratio than the «stable cognitive group», which indicated a higher frequency of playing mahjong in the «high initial level - cognitive decline group».
Lee et al. (2020)	2012, 2014	aged 65 or above	4718	Playing mahjong	Sleep	• Sleep quality • Average hours of sleep daily • Predictors (1. group social activity and 2. playing the Mahjong card game)	• Older adults who played mahjong card game daily reported a higher prevalence of good-quality sleep than those who participated less frequently. Compared with daily participation, older adults who did not play mahjong reported lower odds of reporting good sleep quality. Weekly participation was negatively associated with sleep quality. • Participation in the Mahjong card game was not associated with the recommended hours of sleep duration.
Teh and Tey (2019)	2005, 2008, 2011	aged 65 or above	15163	Playing mahjong or cards	Loneliness	• Frequency of leisure activities • Loneliness	• No significant effects were observed in the 2008 wave.
Gao et al. (2018)	2005, 2008, 2011	aged 65 or above	104468	Playing mahjong or cards	Functional disability	• Functional disability • Age of onset of ADL disability • Frequency of social participation • Mediators (Physical exercise, Positive emotions, MMSE)	• Compared with those who never played cards/mahjong, older adults who frequently and occasionally played cards /mahjong were significantly less likely to feel persistently lonely. Compared with the baseline in 2005, older adults who frequently played cards / mahjong reported significantly lower odd ratios of persistent loneliness in 2011. No significant effect was observed for the occasional players. • Older adults who never played cards / mahjong showed a more rapid decline of ADL than those who have frequent participation. Playing mahjong/cards reduced the risk of having an incident functional disability. • Cognitive ability, positive emotions and physical exercise were the significant mediators of the relationship between playing cards / mahjong and functional disability. Cognitive ability was the strongest mediator.
Qiu et al. (2019)	1998, 2000, 2002, 2005, 2008, 2011, 2014	aged 80 or above	4830	Playing mahjong or cards	Cognitive function	• MMSE • Frequency of cognitive leisure activities	• Compared with older adults who «never» played cards / mahjong, those who participated «sometimes» or» almost every day» did not show a significant trend. • Sometimes playing cards or mahjong were associated with a decreased risk of cognitive impairment.
Mao et al. (2020)	1998, 2000, 2002, 2005, 2008, 2011	aged 80 or above	10741	Playing mahjong or cards	Cognitive function	• Chinese version of MMSE • Frequency of leisure activity • Depressive symptoms	• Compared with older adults who «never» played cards / mahjong, those who participated «almost every day» and «sometimes» showed significantly lower hazard ratios. • Compared with those who «never» engaged in playing cards or mahjong, the estimated effects of engaging in these activities «sometimes» or «almost every day» showed a significantly reduced risk of cognitive impairment in the octogenarians and nonagenarians, but not in the centenarians. An interaction between the frequencies «sometimes» and «never» was found, indicating that older adults with more than two years of education have a lower hazard ratio.
Ni et al. (2020)	2002, 2005, 2008, 2011, 2014	aged 60 or above	1314	Playing mahjong	Negative affect and cognitive function	• MMSE • Negative affect	During the 5 waves, mahjong players were associated with slower rates of negative affect increase and cognitive decline.
Zhao and Li (2022)	2002, 2005, 2008–09, 2011–12, 2014, 2017–18	aged 65 or above	2406	Playing mahjong or cards	Cognitive function, subjective well-being and IADL	• Playing cards or mahjong (frequency) • IADL • MMSE • Negative subjective well-being	• Frequent participation in playing mahjong or cards buffered the detrimental effect of widowhood on IADL abilities in both men and women. • No significant interaction effects between widowhood and playing mahjong were found on cognitive ability and negative subjective well-being.
Zhang et al. (2023)	2002, 2005, 2008, 2011, 2014	aged 65 or above		Playing mahjong or cards	Cognitive function	• Chinese version of MMSE • Frequency of leisure activity (yes/no)	• Playing cards / mah-jong was associated with decreased probabilities of cognitive impairment at the next wave. • Playing cards / mah-jong was associated with reduced probabilities of cognitive impairment.
Wang et al. (2022)	2002–2005, 2005–2008, 2008–2011, 2011–2014	aged 65 or above	12280	Playing mahjong or cards	Cognitive function	• Leisure activity (frequency) • MMSE	• Compared with older adults who «rarely or never» played cards / mahjong, those who played mahjong showed a decreased risk of cognitive impairment. • Compared with those whose behavior did not change, the associations of playing less mahjong or cards were more likely to develop cognitive impairment. • Compared with individuals who did not change the frequency of playing mahjong or cards, those who played a little bit more or played much more decreased the risk of developing cognitive impairment.
Sha et al. (2022)	2002, 2005, 2008, 2011, 2014	aged 65 or above	7422	Playing mahjong or cards	Cognitive function	• Chinese version of MMSE • Playing cards or mahjong (frequency)	• Playing cards or mah-jongg daily were associated with higher possibility of reversion than those who occasionally or never did them. • Further restriction on MMSE change (≥2 and ≥3 points) to the definition of reversion, daily playing mah-jongg or other card games continued to be significantly associated with reversion.
Ren et al. (2023)	2014, 2018	aged 65 or above	ADL: 6047 IADL: 6216 Cognitive function: 5916	Playing mahjong or cards	Cognitive function, ADL and IADL	• Leisure activity (frequency) • ADL • IADL • Chinese version of MMSE	• Playing cards or mahjong was correlated with a 33.1% decreased risk of cognitive impairment. • No significant effects were found on ADL and IADL disability.
Tian et al. (2022)	2008, 2011, 2014, 2018	aged 65 or above	11821	Playing mahjong or cards	Cognitive function	• Playing cards or mahjong (frequency) • MMSE	• With the increase in playing cards / mahjong frequency, the crude rate of dementia events decreased gradually. • Compared with participants who rarely or never played cards/mahjong, participants who played cards/mahjong almost every day had a significantly lower risk of dementia. • Similar results were found in subgroup analyses based on sex, age, regular exercise and MMSE score.

Note. MMSE = Mini-Mental State Examination; ADL = activities of daily living; IADL = Instrumental activities of daily living.

All of these 16 studies used secondary data from the Chinese Longitudinal Healthy Longevity Survey, which is a large-scale population-based study that started in 1998. The survey examines factors related to healthy longevity using face-to-face interviews conducted every two to three years. The number of waves included in the 16 studies ranged from two to seven. Most of these studies (n = 12, 75%) examined the relationship between long-term participation in mahjong and the risk of cognitive, psychological, and functional decline (6, 13, 23, 24, 37–44). Three of the studies (18.8%) examined the factors influencing cognitive trajectories (12, 45, 46), and one pioneering study (6.3%) investigated the possibility of reversion from MCI (47). Most of the studies focused on cognitive outcomes (n = 10; 62.5%). Only three of the studies (18.8%) focused on psychological and functional outcomes, such as loneliness (13), functional disability (37), and sleep quality (38). Three studies (18.8%) examined cognitive function concurrently with negative affect (40), ADL and instrumental activities of daily living (IADL; 44), and subjective well-being and IADL (24).

Playing mahjong was found to be associated with a reduced risk of cognitive impairment or dementia (6, 23, 39, 41, 43, 44), better cognitive functioning (42, 46), and relatively stable or slow cognitive decline (12, 40, 45). Daily participation in mahjong was found to be associated with a higher possibility of reversion from MCI (47) and frequently playing the game decreased the risk of developing cognitive impairment (43). Another study reported that occasional participation was associated with a decreased risk of cognitive impairment (41). Compared with older adults who did not participate in mahjong, those who played the game were less likely to experience persistent loneliness (13) and demonstrated a slower rate of negative affect increase (40). They also exhibited a lower risk of incident functional disability (37) and reported better sleep quality (38). However, two studies found no significant relationship between playing mahjong and the focal outcomes, namely cognitive functioning (24), subjective well-being (24), and ADL and IADL (44).

### Playing Mahjong and Disease Prevalence

Eleven cross-sectional (20.8%) and four case-control studies (7.5%) examined whether playing mahjong was associated with the prevalence of clinical conditions and factors related to clinical conditions. Table 3 summarizes the findings of the cross-sectional and case-control studies.

**Table 3:** Data Charting on Associations between Playing Mahjong and Disease Prevalence (n = 11) and the Case-Control Studies (n =4)

**Study**	**Designs**	**Disease/ Construct**	**Activity**	**N**	**Main outcomes**	**Key findings**
Wang et al. (2017a)*	Cross-sectional	Prevalence of MCI	Playing chess, cards, or mahjong	1781	MMSE, IADL, GDS	Older adults who played mahjong/chess/cards showed a significantly different rate of MCI prevalence, in which playing mahjong every day was a protective factor for MCI compared to no participation.
Cao et al. (2017a)	Cross-sectional	Prevalence of cognitive impairment	Playing chess, cards, or mahjong	84	MMSE, IADL, GDS	Older adults who played mahjong/chess/cards did not show a significantly different rate of cognitive impairment prevalence.
Cao et al. (2017b)	Cross-sectional	Prevalence of dementia	Playing chess, cards, or mahjong	84	MMSE, IADL, GDS	Older adults who played mahjong/chess/cards showed a significantly different rate of dementia prevalence, in which playing mahjong at least once a week was a protective factor against dementia compared to no participation.
Wang et al. (2020)	Cross-sectional	Prevalence of Parkinsonism	Playing mahjong or cards	3996	PDSI	• Older adults who played mahjong / cards showed a significantly different rate of Parkinsonism prevalence, in which always playing mahjong/cards was a protective factor against Parkinsonism compared to no participation. • Seldom playing mahjong/cards showed no significant effects.
Wang et al. (2017b)*	Cross-sectional	Prevalence of MCI	Playing chess, cards, or mahjong	84	MMSE, IADL, GDS	Older adults who played mahjong/chess/cards showed a significantly different rate of MCI prevalence, but it was neither a protective nor risk factor for MCI.
Cai et al. (2020)	Cross-sectional	Prevalence of ADL impairment	Playing mahjong or cards	3978	IADL	• Older adults who played mahjong/cards showed a significantly different rate of ADL impairment prevalence, in which always playing mahjong was a protective factor against ADL impairment compared to no participation. • Seldom playing mahjong/cards showed no significant effects.
Guo et al. (2020)	Cross-sectional	Prevalence of cognitive impairment	Playing mahjong or cards	3996	AD 8, MMSE	Older adults who played mahjong/cards showed a significantly different rate of cognitive impairment prevalence, in which always and seldom playing mahjong/cards was a protective factor against cognitive impairment compared to no participation.
Ayijiamali et al. (2015)	Cross-sectional	Prevalence of depression	Playing mahjong	1329	GMS	Playing mahjong was a protective factor against depression compared to no participation.
Cao et al. (2017c)*	Cross-sectional	Prevalence of dementia	Playing chess, cards, or mahjong	415	MMSE, IADL, GDS	Older adults who played mahjong/chess/cards did not show a significantly different rate of dementia prevalence.
Deng et al. (2018)*	Cross-sectional	Prevalence of dementia	Playing mahjong or chess	1781	MMSE, IADL, GDS	Older adults who played mahjong / chess showed a significantly different rate of dementia prevalence, but it was neither a protective nor risk factor for dementia.
Tang et al. (2021)	Cross-sectional	Prevalence of depression	Playing mahjong or cards	19420	PHQ-9, MMSE	Older adults who played mahjong or cards showed a significantly different rate of depression prevalence, in which playing mahjong or cards usually was a protective factor for depression compared to seldom participation.
Qi et al. (2018)	Case-control	Factors related to cognitive impairment	Playing mahjong or cards	1300 cognitive impairment, 2600 healthy controls	AD 8, MMSE	• There was a significant difference in the frequency of playing mahjong/cards between the control and people with cognitive impairment. • Compared to older adults who never played mahjong/cards, always and seldom playing mahjong/ cards was a significant protective factor against cognitive impairment.
Shi et al. (2012)	Case-control	Factors related to AD	Playing chess, cards, or mahjong	78 AD, 156 healthy controls	MMSE, CDR	Playing chess /cards/mahjong was a significant protective factor against AD.
Xue et al. (2012)	Case-control	Factors related to MCI	Playing chess, cards, or mahjong	84 MCI, 168 healthy controls	MMSE, CDR	Playing chess /cards/mahjong was a significant protective factor against MCI.
Sun et al. (2023)	Case-control	Factors related to cognitive impairment	Playing mahjong or cards	1300 cognitive impairment, 2600 healthy controls	AD 8, MMSE	• There was a significant difference in the behaviour of playing mahjong/cards between the control and people with cognitive impairment in APOE e4 carrier and noncarrier. • Playing cards or mahjong was a significant protective factor against cognitive impairment in APOE carriers and noncarriers.

Note. All of the 11 cross-sectional studies were analysed by Chi-square and logistic regression except Ayijiamali et al. (2015) using the Chi-square test only. All of the 11 cross-sectional studies and 4 case-control studies were analysed by univariate and multivariate regression. All of the 11 cross-sectional studies and 4 case-control studies recruited older adults aged 60 years or above. Protective factors were indicated by odd ratios larger than 1 while risk factors were indicated by odd ratios smaller than 1. MCI = Mild cognitive impairment; ADL = Activities of daily living; MMSE = Mini-Mental State Examination; IADL = Instrumental Activities of Daily Living; GDS = Geriatric Depression Scale; GMS = Geriatric Mental State Schedule; PDSI = Parkinson’s disease symptom inventory; AD8 = Chinese version of 8-item Ascertain Dementia; PHQ-9 = Patient Health Questionnaire-9; AD = Alzheimer’s disease; CDR = Clinical Dementia Rating; APOE = Apolipoprotein E.

* Studies that have specified recruiting healthy older adults in the methodology

Most of the 11 cross-sectional studies (n = 7; 63.6%) focused on cognitive disorders such as cognitive impairment (48, 49), MCI (50, 51), and dementia (52–54). Four of the studies (36.4%) focused on psychological and functional disorders such as depression (55, 56), Parkinsonism (57), and ADL impairment (58).

Most of the 11 cross-sectional studies (n = 9, 81.8%) reported significant differences in mahjong participation in terms of disease prevalence (49–52, 54–58). Two of the studies (18.2%) found no significant differences between those who participated in mahjong and those who did not in the prevalence of cognitive impairment (48) and dementia (53).

Additional analyses focused on ascertaining whether playing mahjong is a risk or protective factor for clinical conditions. In seven of the studies (77.8%), playing mahjong was found to be a protective factor against various clinical conditions, namely cognitive impairment (49), MCI (51), dementia (52), Parkinsonism (57), ADL impairment (58), and depression (55, 56). Two of the studies (22.2%) reported no significant effects on MCI (50) and dementia (54).

The four case-control studies that probed the factors pertaining to cognitive-related disorders examined cognitive impairment (41, 59), MCI (60), and Alzheimer’s disease (61). After matching the controlled subjects on sex, age, and residential area, all of them reported that playing mahjong was a significant protective factor against the focal clinical conditions. Sun et al. (2023) reported that the protective effect was similar for carriers and noncarriers of the APOE gene.

### Effectiveness of Mahjong Interventions

Six intervention studies, including four RCTs and two non-RCTs, examined the effects of mahjong interventions on different populations and across different outcomes. Table 4 summarizes their designs and findings and Table S3 presents their effect sizes.

**Table 4:** Data Charting on Intervention Studies (n = 6)

**Study**	**Country**	**Designs**	**Population**	**N**	**Construct(s)**	**Main outcomes**	**Key findings**
Zhang et al. (2020)	China	RCT	MCI	• 28 mahjong intervention • 28 passive control	• executive function • IADL	• MoCA-B • STT • FAQ	Playing mahjong significantly improved executive functions and IADL over time, but the differences were not observed in the control group.
Cheng et al. (2006)*	Hong Kong	non-RCT	Dementia	• 29 (4X) • 33 (2X)	• cognitive functioning (digit forward memory, verbal memory, MMSE)	• MMSE • the Chinese Digit Span Test - digit forward span and sequence • the Chinese Auditory Verbal Learning Test	There was no significant difference between the 2X and 4X groups, which suggested similar benefits for playing twice and playing four times a week.
Cheng et al. (2014b)	Hong Kong	RCT	Dementia	• 36 mahjong intervention • 39 tai chi • 35 handicrafts	• cognitive and functional deterioration	• CDR sum-of-box score	Compared with control, playing mahjong showed a slower progression over time.
Lu et al. (2015)	Taiwan	non-RCT	Healthy	• 45 mahjong intervention • 47 passive control	• short-term memory • attention • logical reasoning	• Corsi Block-Tapping Test • Focused Attention Test • Raven’s Coloured Progressive Matrices Test	Playing mahjong significantly improved short-term memory, attention and logical reasoning capacity.
Cheng et al. (2012)	Hong Kong	RCT	Dementia	• 12 mahjong intervention • 12 tai chi • 12 handicrafts	• depression	• GDS	Playing mahjong showed. immediate benefits on depression but the effect was reverted to the baseline level at the follow-up.
Cheng et al. (2014a)	Hong Kong	RCT	Dementia	• 36 mahjong intervention • 39 tai chi • 35 handicrafts	• cognitive functioning (MMSE, digit forward and backward memory, verbal and episodic memory, semantic memory)	• MMSE • Digit forward memory - span and sequence • Digit backward memory - span and sequence •Verbal memory - immediate and delayed recall • Categorical verbal fluency	Playing mahjong showed long-term benefits in the short-term memory of numerical units.

Note. All assessments were assumed using the Chinese version. RCT = randomized controlled trial; MCI = Mild Cognitive Impairment; IADL = Instrumental Activities of Daily Living; MoCA-B = the Montreal Cognitive Assessment Scale—Beijing; STT = Shape Trial Test; FAQ = Functional Activities Questionnaire; MMSE = Mini-Mental State Examination; 4X = four times a week; 2X = two times a week; GDS = Geriatric Depression Scale; CDR = Clinical Dementia Rating. * This study examined playing mahjong twice or four times a week for 16 weeks. The remaining studies examined mahjong intervention for 12 weeks.

Five of the studies (83.3%) examined the effects of a 12-week mahjong intervention in which participants played an hour of mahjong three times a week. Only one study (16.7%), which investigated the effects of a 16-week mahjong intervention, manipulated the frequency of playing (8). All of the studies involved at least one control group that participated either passively or actively by engaging in different activities such as tai chi or handicrafts.

Most of the intervention studies (n = 5; 83.3%) recruited clinical populations with cognitive impairments such as MCI and dementia. One study (16.7%), conducted in Taiwan, included healthy populations (62). Most of the studies (n = 5; 83.3%) focused on cognitive functioning aspects such as general cognition, digit span and verbal memory, executive function, attention, and reasoning (8, 10, 20, 62, 63). Aspects of psychological functioning [specifically depressive symptoms (11); and functional independence (63)] were the least studied.

All of the studies revealed that a mahjong intervention lasting for at least 12 weeks resulted in significant positive benefits in terms of cognitive, psychological, and functional outcomes. The interventions improved general cognitive abilities, with small to large effect sizes, as measured by the MMSE (d = 0.77 [medium]), MoCA (d = 1.26 [large]), and the sum-of-boxes of the Clinical Dementia Rating scale (d = −0.34 [small]) (10, 20, 63). They also enhanced performance, with effect sizes ranging from small to large, in cognitive tasks such as the Forward Digit Span Task (d = 0.58 [medium]), Forward Digit Sequencing Task (d = 0.69 [large]), Corsi Block-Tapping Test (immediate block span; d = 0.62 [medium]), and Focused Attention Tests (average reaction time; d = −0.2 [small]) (20, 62). The interventions also improved higherorder cognitive abilities, such as logical reasoning and executive function, measured using Raven’s Colored Progressive Matrices Test (d = 2.02 [large]), Categorical Fluency Test (d = 0.27 [small]), and the Shape Trail Test (d = −1.43 [large]) with either small or large effect sizes (20, 62, 63). Improvements with medium to large effect sizes were also observed in psychological and functional outcomes upon the completion of the mahjong interventions, as measured by the Geriatric Depression Scale (d = −0.57 [medium]) (11) and the Functional Activities Questionnaire (d = −1.31 [large]) (63), respectively. However, the intervention did not improve scores in verbal recall and the Backward Digit Span and Backward Digit Sequencing Tasks (20).

## Discussion

This review scoped the literature on playing mahjong across Western and Asian databases, aiming to identify research gaps and provide directions for future research. Consistent with expectations, both the observational and intervention studies supported the idea that playing mahjong is a beneficial leisure activity. Cheng et al. (2006) suggested that the complex game rules and social nature of mahjong promote cognitive, psychological, and functional abilities. The review systematically classified the areas explored in the literature on mahjong into five major themes: subjective meaning attached to playing mahjong, short-term benefits of playing mahjong, long-term benefits of playing mahjong, association between playing mahjong and disease prevalence, and the effectiveness of mahjong interventions. Most of the studies on the subject adopted a correlational analysis approach. Only a few of them were RCTs, indicating that more high-quality RCTs are necessary to establish the utility of mahjong in improving various outcomes.

### Qualitative Findings on the Benefits of Playing Mahjong

The qualitative evidence supported the proposition that playing mahjong has cognitive and psychological benefits. The qualitative studies used a bottom-up approach to explore the meaning of playing mahjong to the older community instead of investigating specific outcomes using scales such as the MMSE, MoCA, or the Geriatric Depression Scale. They complemented the studies that used questionnaires and explained the benefits of playing mahjong by revealing that it helped older adults gain a sense of self-esteem and competence, alleviated their worries, and facilitated greater social support. An interesting explanation of the value that playing mahjong provided to the older adults, revealed in one of the studies, was that it stimulated a sense of youthfulness. This finding suggested that playing mahjong not only enhanced performance in cognitive and psychological tests but also fostered a positive self-concept. A positive outlook on aging increases self-efficacy in health, which further reinforces a health-promoting lifestyle (64). The positive feedback obtained from playing mahjong may help to cultivate psychological health and encourage a positive aging experience.

These studies discussed the benefits of playing mahjong from a qualitative viewpoint. Interviewing the older adults revealed how and why playing mahjong is effective. However, they did not clarify which aspects of health and well-being it improved and the extent of those improvements. Whereas the qualitative findings revealed the benefits of playing mahjong at a conceptual level, they did not explain the mechanisms underlying these effects or the importance of each contributing factor. Additional research is necessary to test the hypotheses generated from the qualitative research, such as by manipulating the identified contributing factors and examining their effects on cognitive, psychological, and functional outcomes.

### Quantitative Findings on the Benefits of Playing Mahjong

Three categories of quantitative findings on the effects of playing mahjong emerged from the observational studies: short-term benefits, long-term benefits, and protective effects on clinical conditions.

The studies that examined the short-term benefits showed that playing mahjong was positively correlated with beneficial outcomes such as improved cognitive functioning, lower psychological distress, and better eye–hand coordination. These correlations may be attributable to the fact that mahjong is a mentally demanding and cognitively stimulating game (8, 10, 20). Players are required to utilize multiple cognitive and social resources during the game, and it therefore offers older adults opportunities to exercise their mental abilities. However, the evidence from these studies was mainly correlational, revealing only the association between playing mahjong and the specific outcomes. They did not explain how playing mahjong led to the beneficial outcomes. Correlational research is generally limited because it provides little insight into causality in relationships. Some of the studies did not find a positive association between playing mahjong and specific outcomes (18, 19). These findings may be due to differences in the frequency of participation in the activity and the inclusion of young older adults. One of these studies measured participation in mahjong with a 9-point scale rather than a dichotomous or trichotomous classification, which may have caused higher variability in the dependent variable and made it more difficult to obtain a significant result (18). In another of these studies, recruiting young older adults, aged 60 to 69 years, may have limited the variation in the outcome, possibly resulting in a lack of statistical significance (19). Therefore, high-quality RCTs that manipulate variables that may affect the outcomes are necessary to complement the findings of the cross-sectional studies.

Another group of the studies adopted a longitudinal design to investigate the long-term benefits by following up on the behavior of older adults who played mahjong. These studies captured changes over time to elucidate the relationship between the habit of playing mahjong and the trajectories of cognitive impairments, patterns of loneliness, and risk of functional disability. Rather than merely using a dichotomous classification of whether one played mahjong or not, examining the frequency of playing enhanced the understanding of how optimal behavioral outcomes could be achieved. For instance, Xue (2020) found that compared with older adults who never played mahjong, those who played mahjong at least once a month exhibited the largest improvements, whereas Mao et al. (2020) found that playing mahjong, whether frequently or infrequently, helped to reduce the risk of cognitive impairment. These inconsistent findings on the optimal playing frequency warrant further investigation. In addition, Wang et al. (2022) studied the change of playing frequency from 2002 to 2014 on the prevention of cognitive impairment and found that playing more could decrease the risk while playing less may increase the risk to develop cognitive impairment. The results may suggest a lifelong practice of playing mahjong may also help to prevent cognitive decline. The results coincided with another cross-sectional study that more than one year of mahjong experience may also help to prevent MCI (33). It seems that a lifelong practice or a lifetime familiarity with mahjong may help to prevent cognitive decline. However, since only two studies on this issue were found, more studies are needed to reach a reliable conclusion.

The studies that examined the relationship between playing mahjong and disease prevalence also reported promising results showing that playing mahjong was associated with fewer cognitive-related disorders and lower depression and functional impairment. The results also demonstrated playing mahjong was a protective factor against those clinical conditions. However, it is possible that the conditions were caused by other co-varying factors such as bodily dysfunction and aging rather than playing mahjong per se. Further investigation through controlled studies is necessary to confirm the relationships reported in these studies.

The three groups of quantitative observational studies predominantly examined the relationship between playing mahjong and cognitive outcomes measured using tests such as the MMSE, MoCA, and short-term memory tests. Only a few of the studies investigated psychological and functional outcomes such as loneliness, psychological well-being, and IADL. Preliminary but limited evidence on the benefits of playing mahjong was found in relation to psychological distress (36) and loneliness (13, 27). More studies are necessary to explore the relationship between playing mahjong and psychological and functional outcomes. A limited number of studies has focused on the effect of playing mahjong across different demographic groups (i.e., age, gender, education, and the years of playing mahjong), more studies are, therefore, needed to understand these potential confounding variables. Future studies should also control these possible confounding factors. The grouping of mahjong with card games and chess in many of the studies may have also had a confounding effect on the findings regarding the benefits of playing mahjong. Understandably, the studies combined these activities due to their similar nature (i.e., they are all cognitively and socially stimulating). Nonetheless, future research should explore playing mahjong as an independent activity and control the covariates to enable clear and concrete conclusions.

### Effectiveness of Mahjong Interventions

The six intervention studies supported the idea that playing mahjong improves cognitive and psychological functioning. Regardless of clinical condition, the mahjong interventions enhanced general cognitive performance and short-term memory and alleviated depressive symptoms in older adults (8, 10, 11, 20, 62, 63). However, only four of the studies were RCTs with standardized study protocols. Because an RCT is the gold standard for evaluating the effectiveness of an intervention, it should be given the highest consideration in the literature. The computed effect sizes of the statistically significant improvements in these studies ranged from small to large, and no apparent pattern could be observed in these effect sizes. The wide range of effect sizes may have reflected the fact that the studies used diverse outcome measures, such as the MMSE, MoCA, and the Clinical Dementia Rating, to assess general cognitive ability. The Digit Span and the Digit Sequencing Tasks were only used in two studies (10, 20). The relationship between playing mahjong and scores on the Forward Digit Span and Forward Digit Sequencing Fasks were significant and exhibited medium effect sizes. However, the relationship between playing mahjong and scores on the Backward Digit Span and Backward Digit Sequencing Tasks were not significant and exhibited negligible effect sizes. Because a mahjong player is required to sort and group the mahjong tiles in consecutive order, the game may enhance the short-term memorization of numerical units. The Backward Digit Span and Backward Digit Sequencing Tasks may measure not just short-term memory but also higher-order cognitive abilities such as working memory. Owing to the diversity of the outcomes used in the studies and the limited amount of evidence that has resulted from them, more studies are needed to clarify and evaluate the effects of mahjong interventions.

By providing emerging evidence of the effects of mahjong interventions, the reviewed studies opened discussion of the mechanisms underlying these effects. Some of the intervention studies defined mahjong as a mentally demanding and cognitively stimulating game and postulated that the intellectual component of playing mahjong may promote cognitive and psychological functioning (8, 10, 20). They reported that playing mahjong resulted in greater improvements in cognitive and psychological functioning than less demanding activities such as Tai Chi and handicrafts (8, 10, 20). Those intervention studies that defined mahjong as a general or social leisure activity also reported similar findings by comparing playing mahjong with a passive control condition (i.e., no intervention) (62, 63). In contrast, the observational studies only indicated a positive relationship between playing mahjong and cognitive, psychological, and functional abilities. It is still not clear which component (i.e., cognitive or social component) contributed to the positive effects of playing mahjong and what the underlying mechanisms are. Therefore, future research could examine the mechanisms underlying the effects of playing mahjong by separating the intellectual and social components and investigating the neural underpinnings of those effects. It would be valuable to clarify whether the positive effects of playing mahjong are due to its nature of being a general leisure activity, an intellectual leisure activity, or a social leisure activity. Future research could also develop the theoretical background on the effects of playing mahjong to foster discussion of whether a cognitively stimulating activity or a socially interactive activity better mitigates age-related decline in older adults. Because aging is associated with brain degeneration and can be measured in neuroimaging studies (65), incorporating neural evidence such as structural and functional brain changes in studies on the effects of playing mahjong may also result in valuable contributions to the aging literature.

Another possible direction could be focusing on the difference between experienced players and novices. The RCTs included in this review recruited participants with MCI or dementia who have already acquired mahjong skills but have not played mahjong regularly in the past three to six months (10, 11, 20, 63). The mahjong skills were acquired many years ago, and it is difficult to separate the effect of familiarity or playing mahjong itself. To better understand the effects of playing mahjong on cognitive abilities in older adults and isolate the effect of familiarity, future studies may investigate the performance between the experienced mahjong player and the newly acquired player.

### Other Directions for Future Work

We propose four additional broad directions that future work could pursue to better understand the benefits of playing mahjong: incorporating diverse outcomes of higher-order functioning, investigating the generalizability of the research findings, examining long-term effects, and studying different populations. The first research direction refers to the further examination of the relationship between playing mahjong and various aspects of higher-order cognitive functioning such as executive function and decision-making. In the reviewed studies, the investigation of the relationship between playing mahjong and general cognitive abilities such as short-term memory and attention was superficial. The MMSE and MoCA were the two major outcomes used in the literature. Although the intervention studies evaluated functioning in specific cognitive domains using tests such as the Digit Span, Corsi Block-Tapping, and Chinese Auditory Verbal Learning Tests, the effects of playing mahjong on higher-order cognitive functioning (e.g., executive function and decision making) remain unknown. Because playing mahjong requires players to remember the game rules and flexibly devise and implement strategies, it might promote working memory and the inhibition and switching aspects of executive functions.

Another potential research direction is to explore whether the findings on the benefits of playing mahjong are generalizable to wider outcomes such as life satisfaction and quality of life. According to a practical handbook for mahjong interventions in Hong Kong (14), the principal objective of a mahjong intervention is to boost happiness and self-confidence among older adults. This objective is consistent with one of the qualitative findings in this scoping review: playing mahjong stimulated a sense of youthfulness and accomplishment and enhanced self-confidence. Future research may extend the scope of this body of research from cognitive, psychological, and functional impact to a broader range of outcomes.

There were also differences in the long-term effects of playing mahjong on cognitive and psychological outcomes. A long-term effect of the activity on cognitive functioning was observed during an one-month follow-up (8) and a six-month follow-up (10, 20). However, its effect on alleviating depressive symptoms was not sustained after three months (11). Due to the limited number of studies, it is difficult to conclude what the long-term effects of playing mahjong actually are. Future studies should not only investigate the long-term effects of playing mahjong but also compare the differences in these effects between different domains of outcomes.

The literature on playing mahjong has hitherto focused on clinical populations such as older adults with dementia, MCI, or depression instead of healthy populations. Because healthy older adults also experience declines in executive function across their lifespan (66), investigating the effects of playing mahjong on healthy older adults could be valuable to develop methods of preventing or slowing age-related declines. Theories on active aging (2) and active participation in leisure activities (39) have suggested that the risk of impairment can be reduced and age-related trajectories can be slowed. RCTs are necessary to examine whether playing mahjong could potentially slow age-related declines.

## Conclusions

The strengths of mahjong as a potential intervention are its cultural popularity and ease of implementation in Asian cultures. It is a simple activity to implement in the community and could minimize age-related decline. Considering that it is a popular activity in Asian societies, this scoping review included both the Western and Asian literature, and it therefore did not overlook the evidence in the Asian literature. It also covered a broad range of study designs and outcomes, and provided a comprehensive overview of the literature on mahjong by highlighting which aspects of the topic necessitate further examination and what types of study designs are required. In light of the convincing evidence on cognitive outcomes, mahjong also presents a unique opportunity to explore the cognitive reserve in older adults. A limitation of this review is that it focused on cognitive, psychological, and functional outcomes, and studies that only examined physical outcomes such as hypertension and mortality were excluded.

In conclusion, this scoping review summarized the positive effects associated with playing mahjong reported in the literature, and the findings supported the idea that the activity benefits older adults. The empirical evidence showed that the activity, which is popular and culturally important in Asian countries, has the potential to ameliorate age-related cognitive, psychological, and functional declines. Considering that most of the studies adopted a correlational research design, more studies adopting other approaches, especially RCTs, are necessary to advance our understanding of the theoretical mechanisms underlying the effects of playing mahjong.

## Supplementary Materials

Supplementary material, approximately 47.4 KB.

## Data Availability

*Availability of data and materials:* All of the source data are available in the cited studies. Extracted data and materials are reported in Table 1 to Table 4 in the manuscript and Table S1 to Table S3 in the supplementary materials. No analytic code is used for the data analysis. Additional information is available upon reasonable request from the first author.
