# Supplementary material for: Does Playing Mahjong Benefit Older Individuals? A Scoping Review
Source: J Prev Alzheimers Dis. 2024 Jun 7;11(5):1363–77. doi: 10.14283/jpad.2024.102 (PMC11436455; doi:10.14283/jpad.2024.102)
Supplement: Supplementary file 1 — Supplementary material, approximately 47.4 KB. [file 42414_2024_102_MOESM1_ESM.docx]

### Supplementary Materials

**Appendix A**

***An Example Search Strategy in Web of Science***

TS=(( mahjong OR mahjongg OR mah-jongg OR "Mah Jong" OR "Pung Chow" OR “tile-based game*” OR tile-based) AND (“older adult*” OR age* OR aging OR elder* OR senior OR “older people” OR “old age*” OR geriatric OR gerontolog* OR senile OR old))

**Appendix B**

***Preferred Reporting Items for Systematic Reviews and Meta-Analyses extension for Scoping Reviews (PRISMA-ScR) Checklist***

| **SECTION** | **ITEM** | **PRISMA-ScR CHECKLIST ITEM** | **REPORTED ON PAGE #** |
| --- | --- | --- | --- |
| **TITLE** | | | |
| Title | 1 | Identify the report as a scoping review. | 1 |
| **ABSTRACT** | | | |
| Structured summary | 2 | Provide a structured summary that includes (as applicable): background, objectives, eligibility criteria, sources of evidence, charting methods, results, and conclusions that relate to the review questions and objectives. | 1 |
| **INTRODUCTION** | | | |
| Rationale | 3 | Describe the rationale for the review in the context of what is already known. Explain why the review questions/objectives lend themselves to a scoping review approach. | 1-2 |
| Objectives | 4 | Provide an explicit statement of the questions and objectives being addressed with reference to their key elements (e.g., population or participants, concepts, and context) or other relevant key elements used to conceptualize the review questions and/or objectives. | 2 |
| **METHODS** | | | |
| Protocol and registration | 5 | Indicate whether a review protocol exists; state if and where it can be accessed (e.g., a Web address); and if available, provide registration information, including the registration number. | 2 |
| Eligibility criteria | 6 | Specify characteristics of the sources of evidence used as eligibility criteria (e.g., years considered, language, and publication status), and provide a rationale. | 2 |
| Information sources* | 7 | Describe all information sources in the search (e.g., databases with dates of coverage and contact with authors to identify additional sources), as well as the date the most recent search was executed. | 2 |
| Search | 8 | Present the full electronic search strategy for at least 1 database, including any limits used, such that it could be repeated. | Supplementary (Appendix A) |
| Selection of sources of evidence† | 9 | State the process for selecting sources of evidence (i.e., screening and eligibility) included in the scoping review. | 2-3 |
| Data charting process‡ | 10 | Describe the methods of charting data from the included sources of evidence (e.g., calibrated forms or forms that have been tested by the team before their use, and whether data charting was done independently or in duplicate) and any processes for obtaining and confirming data from investigators. | 2-3 |
| Data items | 11 | List and define all variables for which data were sought and any assumptions and simplifications made. | 3 |
| Critical appraisal of individual sources of evidence§ | 12 | If done, provide a rationale for conducting a critical appraisal of included sources of evidence; describe the methods used and how this information was used in any data synthesis (if appropriate). | N/A |
| Synthesis of results | 13 | Describe the methods of handling and summarizing the data that were charted. | 3 |
| **RESULTS** | | | |
| Selection of sources of evidence | 14 | Give numbers of sources of evidence screened, assessed for eligibility, and included in the review, with reasons for exclusions at each stage, ideally using a flow diagram. | 3 |
| Characteristics of sources of evidence | 15 | For each source of evidence, present characteristics for which data were charted and provide the citations. | 3-10 |
| Critical appraisal within sources of evidence | 16 | If done, present data on critical appraisal of included sources of evidence (see item 12). | N/A |
| Results of individual sources of evidence | 17 | For each included source of evidence, present the relevant data that were charted that relate to the review questions and objectives. | 3-10 |
| Synthesis of results | 18 | Summarize and/or present the charting results as they relate to the review questions and objectives. | 3-10 |
| **DISCUSSION** | | | |
| Summary of evidence | 19 | Summarize the main results (including an overview of concepts, themes, and types of evidence available), link to the review questions and objectives, and consider the relevance to key groups. | 9-12 |
| Limitations | 20 | Discuss the limitations of the scoping review process. | 13 |
| Conclusions | 21 | Provide a general interpretation of the results with respect to the review questions and objectives, as well as potential implications and/or next steps. | 13 |
| **FUNDING** | | | |
| Funding | 22 | Describe sources of funding for the included sources of evidence, as well as sources of funding for the scoping review. Describe the role of the funders of the scoping review. | 13 |

JBI = Joanna Briggs Institute; PRISMA-ScR = Preferred Reporting Items for Systematic reviews and Meta-Analyses extension for Scoping Reviews.

* Where *sources of evidence* (see second footnote) are compiled from, such as bibliographic databases, social media platforms, and Web sites.

† A more inclusive/heterogeneous term used to account for the different types of evidence or data sources (e.g., quantitative and/or qualitative research, expert opinion, and policy documents) that may be eligible in a scoping review as opposed to only studies. This is not to be confused with *information sources* (see first footnote).

‡ The frameworks by Arksey and O’Malley (6) and Levac and colleagues (7) and the JBI guidance (4, 5) refer to the process of data extraction in a scoping review as data charting*.*

§ The process of systematically examining research evidence to assess its validity, results, and relevance before using it to inform a decision. This term is used for items 12 and 19 instead of "risk of bias" (which is more applicable to systematic reviews of interventions) to include and acknowledge the various sources of evidence that may be used in a scoping review (e.g., quantitative and/or qualitative research, expert opinion, and policy document).

*From:* Tricco AC, Lillie E, Zarin W, O'Brien KK, Colquhoun H, Levac D, et al. PRISMA Extension for Scoping Reviews (PRISMAScR): Checklist and Explanation. Ann Intern Med. 2018;169:467–473. [doi: 10.7326/M18-0850](http://annals.org/aim/fullarticle/2700389/prisma-extension-scoping-reviews-prisma-scr-checklist-explanation).

**Table S1**

*Overview of the Studies Characteristics (n = 53)*

| Variable | | Number of studies | Percentage of studies |
| --- | --- | --- | --- |
| Country of the study | |  |  |
|  | China | 43 | 81.1% |
|  | Hong Kong | 7 | 13.2% |
|  | Taiwan | 2 | 3.8% |
|  | New York | 1 | 1.9% |
| Language of the literature | |  |  |
|  | Chinese | 23 | 43.4% |
|  | English | 30 | 56.6% |
| Type of study | |  |  |
|  | Observational | 47 | 88.7% |
|  | Intervention | 6 | 11.3% |
| Study design | |  |  |
|  | Qualitative interviews | 3 | 5.7% |
|  | Cross-sectional | 24 | 45.3% |
|  | Longitudinal | 16 | 30.2% |
|  | Case-control | 4 | 7.5% |
|  | Randomized controlled trial | 4 | 7.5% |
|  | Non-randomized controlled trial | 2 | 3.8% |
| Definition of mahjong | |  |  |
|  | General leisure activity | 30 | 56.6% |
|  | Intellectual leisure activity | 10 | 18.9% |
|  | Social leisure activity | 6 | 11.3% |
|  | Intellectual and social leisure activity | 7 | 13.2% |
| Independent variable | |  |  |
|  | Playing mahjong | 17 | 32.1% |
|  | Playing mahjong or cards | 27 | 50.9% |
|  | Playing mahjong or chess | 1 | 1.9% |
|  | Playing mahjong, cards or chess | 8 | 15.1% |
| Outcomes | |  |  |
|  | Cognitive | 31 | 58.5% |
|  | Psychological | 10 | 18.9% |
|  | Functional | 5 | 9.4% |
|  | Cognitive and Psychological | 3 | 5.7% |
|  | Cognitive and Functional | 2 | 3.8% |
|  | Psychological and Functional | 0 | 0% |
|  | Cognitive, Psychological and Functional | 2 | 3.8% |

**Table S2**

*Data Charting on Qualitative Studies (n = 3)*

| Study | *N* | Data analysis | Theme(s) | Categories and Findings |
| --- | --- | --- | --- | --- |
| Kim (2020) | 14 | Thematic analysis | Healthy mahjong | To socialize, exercise the brain, and pass the time |
| Liu (2015) | 42 | Narrative analysis | Individual emotional or material satisfaction | To focus, get rid of worries, kill time, gain happiness, a sense of self-esteem and being young, and monetary reward |
|  |  |  | Reciprocal societal support | To escape from loneliness and depression, also providing social support and “saving face” |
|  |  |  | Lack of family support | To escape from the housework and being lazy |
| Wu and Tang (2011) | 9 | Grounded theory | Emotional support | To provide emotional support between the players via team building and social communication |
|  |  |  | Meaningful participation | Mahjong is an active activity compared with watching television, also stimulates a feeling of youthfulness, and offers mental training opportunities. |
|  |  |  | Satisfaction | To gain a sense of accomplishment and self-confidence from winning the game |

**Table S3**

*Effect sizes for Intervention Studies*

| Study | Outcomes | Metrics extracted from the study | | | Converted to Cohen’s *d* | | |
| --- | --- | --- | --- | --- | --- | --- | --- |
|  |  | Group | Time | Interaction | Group | Time | Interaction |
| Cheng et al. (2006) | MMSE | N/A | Baseline to post-test:   - 2X: *d* = 0.58 - 4X: *d* = 0.58   Baseline to 1-month follow-up:   - 2X: *d* = 0.49 - 4X: *d* = 0.66 | N/A | N/A | N/A | N/A |
|  | Digit Forward Span | N/A | Baseline to post-test:   - 2X: *d* = 1.36 - 4X: *d* = 0.98   Baseline to 1-month follow-up:   - 2X: *d* = 1.33 - 4X: *d* = 1.56 | N/A | N/A | N/A | N/A |
|  | Digit Forward Sequence | N/A | Baseline to post-test:   - 2X: *d* = 1.48 - 4X: *d* = 0.97   Baseline to 1-month follow-up:   - 2X: *d* = 1.39 - 4X: *d* = 1.56 | N/A | N/A | N/A | N/A |
|  | Auditory Verbal Learning Test (trial 1) | N/A | Baseline to post-test:   - 2X: *d* = 0.52 - 4X: *d* = 0.92   Baseline to 1-month follow-up:   - 2X: *d* = 1.06 - 4X: *d* = 1.06 | N/A | N/A | N/A | N/A |
|  | Auditory Verbal Learning Test (trial 2) | N/A | Baseline to post-test:   - 2X: *d* = 0.25 - 4X: *d* = 0.47   Baseline to 1-month follow-up:   - 2X: *d* = 0.73 - 4X: *d* = 0.72 | N/A | N/A | N/A | N/A |
|  | Auditory Verbal Learning Test (trial 3) | N/A | Baseline to post-test:   - 2X: *d* = 0.15 - 4X: *d* = 0.15   Baseline to 1-month follow-up:   - 2X: *d* = 0.68 - 4X: *d* = 0.65 | N/A | N/A | N/A | N/A |
|  | Auditory Verbal Learning Test (trial 4) | N/A | Baseline to post-test:   - 2X: *d* = 0.04 - 4X: *d* = 0.03   Baseline to 1-month follow-up:   - 2X: *d* = 0.10 - 4X: *d* = 0.44 | N/A | N/A | N/A | N/A |
|  | Auditory Verbal Learning Test (trial 5) | N/A | Baseline to post-test:   - 2X: *d* = -0.15 - 4X: *d* = 0.25   Baseline to 1-month follow-up:   - 2X: *d* = 0.32 - 4X: *d* = 0.40 | N/A | N/A | N/A | N/A |
| Cheng et al. (2012) | GDS^*^ | vs. control: *d* = -1.06  vs. Taichi: *d* = -0.68 | *d* = -0.82 |  | -1.06 | -0.82 | -0.57 |
| Cheng et al. (2014a) | MMSE | *B* = 2.24 (0.36, 4.12) | N/A | *B* = 1.48 (0.83, 2.12) | 0.40 | N/A | 0.77 |
|  | Digit Forward Sequence | *B* = 0.69 (0.06, 1.04) | N/A | *B* = 0.66 (0.34, 0.98) | 0.48 | N/A | 0.69 |
|  | Digit Backward Sequence | *B* = 0.47 (-0.04, 0.98) | N/A | *B* = 0.08 (-0.19, 0.35) | 0.31 | N/A | 0.10 |
|  | Digit Forward Span | *B* = 0.78 (0.19, 1.36) | N/A | *B* = 0.67 (0.28, 1.06) | 0.45 | N/A | 0.58 |
|  | Digit Backward Span | *B* = 0.62 (-0.04, 1.28) | N/A | *B* = 0.19 (-0.15, 0.53) | 0.32 | N/A | 0.19 |
|  | Verbal memory – immediate recall | *B* = 0.31 (-0.03, 0.65) | N/A | *B* = 0.13 (-0.08, 0.33) | 0.31 | N/A | 0.21 |
|  | Verbal memory – delayed recalled | *B* = 0.21 (-0.96, 1.38) | N/A | *B* = 0.37 (-0.03, 0.77) | 0.06 | N/A | 0.31 |
|  | Categorical Fluency | *B* = 3.46 (1.02, 5.90) | N/A | *B* = 1.13 (-0.29, 2.54) | 0.48 | N/A | 0.27 |
| Cheng et al. (2014b) | CDR - sum-of-box^*^ | *B* = -0.33 (-2.75, 2.1) | N/A | *B* = -0.45 (-0.88, -0.02) | -0.04 | N/A | -0.34 |
|  | CDR – cognition^*^ | *B* = 0.43 (-1.51, 2.35) | N/A | *B* = -0.09 (-0.33, 0.15) | 0.07 | N/A | -0.12 |
|  | CDR – functioning^*^ | *B* = -0.66 (-1.59, 0.28) | N/A | *B* = -0.06 (-0.30, 0.19) | -0.23 | N/A | -0.08 |
| Lu et al. (2015) | STM - immediate block span | *β* = -0.05, *SE* = 0.05 | N/A | *β* = 0.3, *SE* = 0.03 | -0.10 | N/A | 0.62 |
|  | STM - correct numbers | *β* = 0.03, *SE* = 0.25 | N/A | *β* = 1.89, *SE* = 0.25 | 0.06 | N/A | 2.33 |
|  | STM - incorrect numbers^*^ | *β* = -0.57, *SE* = 0.31 | N/A | *β* = -1.02, *SE* = 0.13 | -1.37 | N/A | -10.04 |
|  | STM - sequencing errors of numbers^*^ | *β* = -0.21, *SE* = 0.3 | N/A | *β* = -0.51, *SE* = 0.16 | -0.42 | N/A | -1.17 |
|  | STM - omitted numbers^*^ | *β* = 1.43, *SE* = 1.1 | N/A | *β* = -3, *SE* = 1.25 | 2.77 | N/A | -2.10 |
|  | Attention - average reaction time^*^ | *β* = 0.02, *SE* = 0.43 | N/A | *β* = -0.1, *SE* = 0.02 | 0.04 | N/A | -0.20 |
|  | Attention - misidentification reaction frequency^*^ | *β* = 1.42, *SE* = 1.47 | N/A | *β* = -4.26, *SE* = 0.59 | 2.79 | N/A | -2.04 |
|  | Attention - neglect reaction frequency^*^ | *β* = 1.8, *SE* = 1.33 | N/A | *β* = -4.23, *SE* = 0.54 | 2.38 | N/A | -2.04 |
|  | Logical reasoning | *β* = -1.18, *SE* = 1.08 | N/A | *β* = 5.27, *SE* = 0.53 | -3.73 | N/A | 2.02 |
| Zhang et al. (2020) | MoCA | N/A | N/A | N/A | 0.44 | 0.81 | 1.26 |
|  | STT^*^ | N/A | N/A | N/A | -0.29 | -0.33 | -1.43 |
|  | FAQ^*^ | N/A | N/A | N/A | -1.01 | -0.48 | -1.31 |
| *Note 1*. MMSE = Mini-Mental State Examination; MoCA = Montreal Cognitive Assessment; GDS = Geriatric Depression Scale; CDR = Clinical Dementia Rating; STM = short-term memory; STT = Shape Trial Test; FAQ = Functional Activities Questionnaire; 4X = four times a week; 2X = two times a week; *B* = unstandardised beta, CI = confidence interval; *β* = standardised beta; SE = standard error; *d* = Cohen’s *d*.  *Note 2*. The *d* (Group) represents the effect size between the treatment (Mahjong) and control group, *d* (Time) represents the effect size between the pretest and posttest of the treatment group, *d* (Interaction) represents the interaction between group and time. The *d* > 0.2, 0.5 and 0.8 are interpreted as small, medium and large effect sizes, respectively.  *Note 3*. Effect sizes were computed and estimated according to Lipsey & Wilson (2001). If the correlation *r* between the pretest and posttest scores was not reported in the article, it was assumed as 0.5 in the computation.  ^*^ Higher score represents poorer performance. | | | | | | | |
